# Parcellation‐based tractographic modeling of the salience network through meta‐analysis

**DOI:** 10.1002/brb3.2646

**Published:** 2022-06-22

**Authors:** Robert G. Briggs, Isabella M. Young, Nicholas B. Dadario, R. Dineth Fonseka, Jorge Hormovas, Parker Allan, Micah L. Larsen, Yueh‐Hsin Lin, Onur Tanglay, B. David Maxwell, Andrew K. Conner, Jordan F. Stafford, Chad A. Glenn, Charles Teo, Michael E. Sughrue

**Affiliations:** ^1^ Department of Neurosurgery University of Oklahoma Health Sciences Center Oklahoma City Oklahoma USA; ^2^ Centre for Minimally Invasive Neurosurgery Prince of Wales Private Hospital Sydney New South Wales Australia; ^3^ Omniscient Neurotechnology Sydney New South Wales Australia; ^4^ Robert Wood Johnson Medical School, Rutgers University New Brunswick New Jersey USA

**Keywords:** anatomy, parcellation, salience network, tractography

## Abstract

**Background:**

The salience network (SN) is a transitory mediator between active and passive states of mind. Multiple cortical areas, including the opercular, insular, and cingulate cortices have been linked in this processing, though knowledge of network connectivity has been devoid of structural specificity.

**Objective:**

The current study sought to create an anatomically specific connectivity model of the neural substrates involved in the salience network.

**Methods:**

A literature search of PubMed and BrainMap Sleuth was conducted for resting‐state and task‐based fMRI studies relevant to the salience network according to PRISMA guidelines. Publicly available meta‐analytic software was utilized to extract relevant fMRI data for the creation of an activation likelihood estimation (ALE) map and relevant parcellations from the human connectome project overlapping with the ALE data were identified for inclusion in our SN model. DSI‐based fiber tractography was then performed on publicaly available data from healthy subjects to determine the structural connections between cortical parcellations comprising the network.

**Results:**

Nine cortical regions were found to comprise the salience network: areas AVI (anterior ventral insula), MI (middle insula), FOP4 (frontal operculum 4), FOP5 (frontal operculum 5), a24pr (anterior 24 prime), a32pr (anterior 32 prime), p32pr (posterior 32 prime), and SCEF (supplementary and cingulate eye field), and 46. The frontal aslant tract was found to connect the opercular‐insular cluster to the middle cingulate clusters of the network, while mostly short U‐fibers connected adjacent nodes of the network.

**Conclusion:**

Here we provide an anatomically specific connectivity model of the neural substrates involved in the salience network. These results may serve as an empiric basis for clinical translation in this region and for future study which seeks to expand our understanding of how specific neural substrates are involved in salience processing and guide subsequent human behavior.

## Graphical abstract

This study provides a detailed model of the salience network (SN) based on its structural and functional connectivity within an anatomically specific parcellation scheme. A coordinate‐based meta‐analysis of the literature and subsequent tractographic analysis on identified regions demonstrate that the SN comprises three clusters of interconnected cortical regions that are extensively connected with both frontal aslant tract fibers and short local association fibers, generally forming a large cingulate and insular‐opercular system. These results may serve as an empirical basis for clinical translation in this region and for future studies that seek to expand our understanding of how specific neural substrates are involved in salience processing and guide subsequent human behavior.

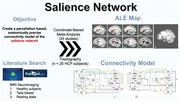

## 1 INTRODUCTION

It has become increasingly clear that information presented to the brain is integrated and processed throughout large‐scale, interacting neural networks to drive subsequent thinking and behavior (Beckmann et al., 2005; Dadario & Sughrue, 2022; De Luca et al., 2006; Thirion et al., 2006). One specific large‐scale brain network, the salience network (SN), is believed to strongly contribute to how the other higher‐order brain networks interact, specifically by allocating cognitive resources between them and initiating appropriate network switching signals according to the type of stimuli presented (Dadario et al., 2021; Menon & Uddin, 2010). As such, the SN has been proposed to serve as a key transitory mediator between passive and active states of mind, and abnormal connectivity within this network is thought to form the underlying basis for a number of neuropsychiatric illnesses (Menon & Uddin, 2010; Seeley et al., 2007).

However, despite the increasing focus of research on the SN since its first characterization (Seeley et al., 2007), there remains relatively little available information on the unique cortical and subcortical connectivity of this network (Seeley, 2019). Such a lack of connectomic information inherently undermines our effective study of the essential functions of the salience system, which is tied to this anatomy, and also limits our understanding of psychopathology or clinical outcomes related to SN disruption (Rosen et al., 2021). Indeed, several studies have improved our understanding of the cortical components of the SN, which is known to activate bilaterally in cortical areas consistent with the anterior insula and frontal operculum, as well as the cingulate gyrus (Menon & Uddin, 2010; Uddin, 2016). While important, existing descriptions of the SN continue to offer limited insight into its underlying structural connectivity and vastly undercharacterize the anatomic structure of the SN at a level of granularity required for precise hypothesis generation and comparison between studies to improve our characterization of this network and for effective clinical translation in these cortices. Newly published surface‐based, multimodal parcellation maps offer a potential remedy to improve the clinical applicability of connectomic models of the SN by providing a highly precise, established cortical atlas and nomenclature within which this network can be reiteratively refined with future work.

The Human Connectome Project (HCP) recently published a surface‐based, multimodal parcellation scheme of the human cerebrum suggesting 180 unique cortical areas within each cerebral hemisphere, which are also believed to be economically connected within organized neural networks (Glasser et al., 2016). Such a detailed, precise atlas provides a common vernacular that allows us to systematically research different cortical regions and neural networks over time and then continually refine these findings in subsequent works with improved data comparisons between studies (Baker, Burks, Briggs, Conner, Glenn, Robbins, et al., 2018; Moreno‐Ortega et al., 2020; Robinson et al., 2010). To provide anatomically precise results within this framework, studies assessing both the functional connectivity of the SN in combination with analyses of its structural interconnectedness may be able to create a precise model of the anatomic substrates of a network in the context of their known functional relevance (Catani et al., 2016; Qi et al., 2012; Ren et al., 2020; Rykhlevskaia et al., 2008). However, focused studies of brain networks have often been limited in the scope of their findings due to their inclusion of highly specific tasks employed in isolation with a small number of subjects that are available. To combat these limitations, other groups, including our own, have begun to apply meta‐analytic software to aggregate large amounts of reported foci in the literature across numerous studies according to predefined search conditions that can target specific networks, such as the SN (Robinson et al., 2010; Turkeltaub et al., 2002). Furthermore, given that many voxelwise studies report their findings in standard stereotaxic space (*x, y, z*), coordinate‐based activation likelihood estimation (ALE) meta‐analyses in particular can analyze the coactivation maps across multiple studies and then assess regions of statistically likely convergence based on the known functions of a specific brain network (Robinson et al., 2010). By utilizing coordinate‐based methodology, one can overcome the heterogeneous topographical nomenclature often described in the literature for homologous brain regions. This methodology has allowed us to precisely characterize many other brain networks in great detail, such as the default mode network (DMN); however, it has not been applied in a similar context for the SN to date and therefore remains a potentially advantageous avenue worth exploring (Milton et al., 2021; Poologaindran et al., 2020; Sandhu et al., 2021; Sheets, Briggs, Dadario, et al., 2021).

Here, we attempted to create a parcellation‐based, anatomically precise cortical model of the SN based on its structural and functional connectivity. Using a collection of open‐access, coordinate‐based meta‐analytic technology to generate ALEs based on healthy functional neuroimaging data, we identified the specific regions of interest (ROIs) likely involved in the SN. Then, we performed structural tractography analyses on these ROIs to determine their structural interconnectedness and the distinct white matter pathways within this network, and we report our findings in the anatomically precise, established HCP parcellation scheme 3 (Glasser et al., 2016). Our goal is to provide a highly detailed connectivity model of the SN in a level of anatomical granularity and specificity that may serve as an empirical foundation to be refined in future studies that assess the essential functions of specific neural substrates of the SN, as well as for clinical translation in associated cortices (Briggs, Allan, et al., 2021; Horn et al., 2017).

## 2 MATERIALS AND METHODS

### 2.1 Literature search

PubMed and BrainMap Sleuth 2.4 (Fox & Lancaster, 2002; Fox et al., 2005; Laird et al., 2005) were queried on August 1, 2017, and again on May 25, 2021, for resting‐state and task‐based functional magnetic resonance imaging (fMRI) studies relevant to the SN according to the Preferred Reporting Items for Systematic Reviews and Meta‐Analyses. Our search strategy included the following algorithm: “salience network OR cingulo‐opercular network AND fMRI.” Studies were included in our analysis if they fulfilled the following search criteria: (a) peer‐reviewed publication, (b) task‐based or resting‐state fMRI study examining the SN, (c) based on whole‐brain, voxelwise imaging, (d) including standardized coordinate‐based results in the Talairach or Montreal Neuroimaging Institute (MNI) coordinate space, and (e) including at least one healthy human control cohort. Only coordinates from healthy subjects were utilized in our analysis. A total of 35 papers met the criteria for inclusion in this study, and the details of these studies are summarized in Table 1. Given that initial searches identified similar anatomical results for both resting‐state and task‐based studies (Smith et al., 2009), our final searches included articles of both natures to improve the power of our findings and were filtered as appropriate.

**TABLE 1:** Summary of the studies included in our meta‐analysis, including the task used, number of participants, what coordinate system was used, and the coordinates that were included to create the Activation Likelihood Estimation. MNI = Montreal Neurological Institute.

Study	Task	Number of participants	MNI/Talairach	Coordinates		
Beaty et al., 2015	Divergent thinking task	25/25 (100%)	MNI	8 ‐36 60	18 4 ‐30	44 ‐4 ‐14
Bhat et al., 2017	Resting‐state (No task)	17/17 (100%)	MNI	‐38 33 ‐17 24 10 5 ‐47 38 ‐23 30 ‐58 55	14 18 8 10 23 14 44 44 44 51 ‐42 ‐42	‐5 ‐4 ‐5 ‐4 23 36 16 16 16 13 29 28
Bilevicius et al., 2017	Resting‐state (No task)	32/32 (100%)	Talairach	21 ‐21 24 ‐60 69 21 ‐20 ‐18	8 ‐40 ‐43 ‐16 ‐37 ‐67 ‐10 ‐49	‐38 4 1 ‐20 14 10 16 ‐29
Chand & Dhamala, 2016	Image categorization task	26/26 (100%)	Talairach	‐6 35 ‐33 4 36 ‐30 ‐21	‐74 9 11 38 ‐47 ‐45 39	‐6 ‐7 ‐8 13 16 ‐10 28
Chen et al., 2016	Resting‐state (No task)	78/78 (100%)	MNI	Session 1 11 55 42 31 48 ‐35 36 37 34 ‐11 ‐1 ‐28 0 5 10 31 26 ‐39 Session 2 11 55 42 31 48 ‐35 36 37 34 ‐11 ‐1 ‐28 0 5 10 31 26 ‐39	‐39 ‐45 0 33 22 20 22 32 16 26 15 52 30 23 22 56 50 51 ‐39 ‐45 0 33 22 20 22 32 16 26 15 52 30 23 22 56 50 51	50 37 47 26 10 0 3 ‐2 ‐8 25 44 21 27 37 27 14 27 17 50 37 47 26 10 0 3 ‐2 ‐8 25 44 21 27 37 27 14 27 17
	Resting‐state (No task)	64/64 (100%)	MNI	‐24 12 39 ‐57 ‐57 ‐6 9 21 ‐27 54	53 ‐73 26 ‐43 ‐52 ‐52 11 8 8 ‐31	4 61 28 49 ‐8 13 40 ‐2 10 25
Doll et al., 2013	Resting‐state (No task)	14/14 (100%)	MNI	39 ‐33 9 ‐6 ‐9 9 33 48 48 ‐45 ‐15 0 ‐48 24	18 9 39 ‐36 ‐21 ‐57 51 ‐45 9 ‐12 ‐30 36 30 ‐3	‐3 ‐6 15 45 6 ‐30 12 30 0 3 ‐6 9 15 ‐15
Elton & Gao. 2014	Global‐local selective task	19/19 (100%)	MNI	‐1 ‐46 47 ‐24 ‐61 ‐59	‐53 ‐64 ‐59 34 ‐35 ‐4	24 24 24 45 ‐7 ‐24
Fang et al., 2016	2‐back task	255/255 (100%)	Talairach	‐6 ‐9 ‐36	15 33 47	42 9 13
Kolesar et al., 2007	Resting‐state (No task)	26/26 (100%)	Talairach	‐6 35 ‐33 4 36 ‐30 ‐21	‐74 9 11 38 ‐47 ‐45 39	‐6 ‐7 ‐8 13 16 ‐10 28
Seeley et al., 2007	Resting‐state (No task)	14/14 (100%)	MNI	42 ‐40 52 ‐52 0 6 ‐6 6 ‐4	10 18 20 16 44 22 18 8 14	‐12 ‐12 ‐18 ‐14 28 30 30 58 48
Sidlauskaite et al., 2014	Cued state‐switching task	18/18 (100%)	MNI	6 ‐4 ‐12 ‐29 ‐36 34 30	35 24 32 24 18 18 24	20 30 24 10 ‐4 6 ‐4
Wang et al., 2016	Resting‐state (No task)	35/35 (100%)	MNI	27 34 ‐2 51 38 9 ‐36 ‐6 0 ‐46 ‐20 14 ‐48 37 ‐12 ‐12 11 32 ‐30 11 ‐30 51 ‐4 54 8 58 43 ‐55 42 ‐41 ‐59 ‐52	49 32 30 23 21 20 18 17 15 10 6 6 6 ‐2 ‐3 ‐12 ‐12 ‐12 ‐14 ‐24 ‐28 ‐30 ‐31 ‐31 ‐40 ‐41 ‐43 ‐44 ‐46 ‐47 ‐47 ‐63	26 7 27 8 ‐1 34 2 34 45 14 7 7 1 ‐3 13 6 6 2 1 2 9 5 ‐4 ‐18 50 20 8 30 21 29 11 15
Haupt et al., 2019	Resting‐state (No task)	32/32 (100%)	MNI	26	46	‐2
Adriana et al., 2019	Resting‐state (No task)	91/91 (100%)	MNI	2 ‐50 ‐44 0 ‐26 0 26	28 ‐60 12 ‐18 40 16 4	22 ‐36 ‐10 50 40 64 26
Hegarty et al., 2020	Isometric muscle contraction during movement tasks	20/20 (100%)	MNI	‐40 ‐1	10 6	‐1 38
Ding et al., 2020	Resting‐state (No task)	35/70 (50%)	MNI	‐6	6	36
Kolesaw et al., 2017	Resting‐state (No task)	14/28 (50%)	Talairach	38 ‐4 ‐52	‐56 22 ‐52	36 39 36
Hernández et al., 2019	Answering questions about a movie which was played in different accents	30/30 (100%)	MNI	6 ‐3 ‐3 ‐63 ‐51 54 42 33 39 57 ‐36 ‐45 ‐39 54 39 ‐30 ‐24 6	32 32 11 ‐28 ‐16 ‐40 50 17 11 ‐10 38 41 47 ‐40 ‐55 ‐70 ‐76 ‐79	17 23 ‐1 20 8 44 8 ‐4 2 5 23 14 14 44 ‐34 ‐19 ‐16 38
Stankewitz et al., 2018	Attentional distraction from painful and nonpainful heat stimulation using a Stroop task	13/26 (50%)	MNI	‐3 6 39 ‐6 3 ‐3	‐9 48 6 ‐45 ‐15 ‐33	63 ‐15 ‐9 0 9 ‐39
Smith et al., 2019	Resting‐state (No task)	17/34 (50%)	Talairach	‐34 ‐65 59 26	31 ‐35 ‐29 ‐83	9 9 12 ‐3
Jarrahi & Mackey, 2018	Resting‐state (No task), imagining a painful condition	15/15 (100%)	MNI	‐38 38 ‐6	6 18 22	‐8 ‐8 32
Bilevicius et al., 2018	Resting‐state (No task)	32/32 (100%)	Talairach	21 ‐21 24 ‐60 69 21 ‐30 ‐18	8 ‐40 ‐43 ‐16 ‐37 ‐67 ‐10 ‐49	‐38 4 1 ‐20 14 10 16 ‐29
De Marco et al., 2017	Resting‐state (No task)	35/35 (100%)	Talairach	26 34 26 32 32 20	‐34 ‐48 ‐51 ‐40 ‐51 ‐30	55 56 63 52 62 59
Zhang et al., 2019	Resting‐state (No task)	20/20 (100%)	MNI	‐9	39	‐3
Conwell et al., 2018	Resting‐state (No task)	45/45 (100%)	MNI	6 38 6 34 50 34 20 32 20 2 ‐20 ‐22 ‐56 56	28 52 26 18 18 26 22 14 8 ‐40 10 10 ‐46 ‐40	28 18 36 4 ‐12 ‐6 8 ‐2 64 48 64 56 48 40
Chou et al., 2017	Resting‐state (No task)	18/35 (51%)	MNI	‐34	14	0
Huang et al., 2020	Resting‐state (No task)	38/72 (53%)	MNI	3	21	24
Jarrahi & Mantini, 2018	Presentation of emotionally salient visual stimuli	33/33 (100%)	MNI	6 30 29 ‐34	20 ‐34 ‐6 21	0 63 ‐2 ‐6
Pang et al., 2021	Resting‐state (No task)	20/20 (100%)	MNI	‐42	15	9
Qiao et al., 2018	AX‐Continuous Performance Task	24/24 (100%)	MNI	9 ‐30 36 ‐30 30	8 23 17 50 50	49 4 4 19 25
Liu et al., 2021	Resting‐state (No task)	19/38 (50%)	MNI	39	48	24
Xin et al., 2021	Resting‐state (No task)	187/197 (95%)	MNI	‐27 30 ‐6 ‐39 42 ‐3	38 41 26 20 14 8	29 29 29 ‐7 ‐4 47
Santangelo & Bordier, 2019	Woking memory task	16/16 (100%)	Talairach	‐42 40 ‐48 48 ‐46 46 ‐32 28 ‐34 36 0 ‐30 24 ‐4 0 50 46 ‐59 0 4 ‐4 4 ‐48 48 ‐2 2 0 4 ‐44	9 13 5 7 ‐3 3 3 9 13 13 ‐6 ‐1 3 ‐6 5 17 15 ‐35 29 21 22 25 15 17 20 20 34 32 15	‐17 ‐19 ‐15 ‐15 ‐13 ‐15 ‐19 ‐21 ‐16 ‐17 ‐8 ‐18 ‐17 ‐8 ‐10 ‐9 ‐7 31 32 41 43 39 ‐9 ‐11 51 51 20 24 ‐9
Lin et al., 2020	Resting‐state (No task)	19/60 (32%)	MNI	‐15 57	24 ‐54	‐3 ‐18

Abbreviation: MNI, Montreal Neurological Institute.

### 2.2 Creation of 3D ROIs

In the original HCP study, parcellation data were analyzed in the CIFTI file format. This is in contrast to traditional file formats, such as NIFTI, which denote regions based on volumetric dimensions (Larobina & Murino, 2014). As a result, it was difficult to perform deterministic tractography using ROIs in CIFTI file format. To convert the parcellation files to volumetric coordinates, the grayordinate label parcellation fields were standardized to the three‐dimensional volumetric working spaces of diffusion spectrum imaging (DSI) Studio (Carnegie Mellon, http://dsi‐studio.labsolver.org) using the structural imaging data provided by the HCP for each subject. This operation was performed utilizing previously created volumetric atlases that convert files from the Connectome Workbench to volumetric working masks in native structural space (Glasser et al., 2013). Specifically, in this pipeline, FreeSurfer annotation files are transformed to each subject's space and then converted to volume masks utilizing a series of FreeSurfer commands we have described elsewhere in great detail (https://surfer.nmr.mgh.harvard.edu/; for exact technical details see CJNeuroLab, 2016; Sheets, Briggs, Young, et al., 2021). Ultimately, this allowed us to convert all 180 parcellations from surface‐based coordinates to volumetric coordinates and thus perform deterministic fiber tractography.

### 2.3 ALE generation and identification of relevant cortical regions

Possible relevant cortical regions in the SN were identified in the literature with methodology reiteratively applied and refined by our team in other regions with great reproducibility (Baker, Burks, Briggs, Conner, Glenn, Sali, et al., 2018; Kuiper et al., 2020; Sandhu et al., 2021; Sheets, Briggs, Dadario, et al., 2021). With software publicly available through BrainMap (http://www.brainmap.org), we used the meta‐analytic software Ginger ALE 2.3.6 to extract the relevant fMRI data for the creation of an ALE (Eickhoff et al., 2012; Turkeltaub et al., 2012). All Talairach coordinates identified during the literature review were converted to the MNI coordinate space using SPM Conversion in Ginger ALE. We subsequently performed a single study analysis using cluster‐level inference in the MNI coordinate space in accordance with common practices and recommendations from original authors (cluster level of 0.05, threshold permutations of 1000, uncorrected *p*‐value of .001 at voxel‐level as the cluster forming threshold; Eickhoff et al., 2012, 2016; Turkeltaub et al., 2002). The ALE coordinate data were displayed on an MNI‐normalized template brain using Multi‐image Analysis GUI (Mango) 4.0.1 (ric.uthscsa.edu/mango). To determine which parcellations should be included in our model, the preconstructed ROIs of the parcellations were overlaid on the ALE and assessed based on the amount of significant overlap. Specifically, the ROI was first placed on the ALE, and then a sphere with the specified volume for each set of coordinates was placed at the ALE coordinate. If a volume of the coordinates was not provided in a study, a default volume sphere with a *r* = 3 mm was utilized. Finally, the *percentage overlap* between the sphere and all overlapping parcel ROIs is calculated. The percentage overlap was calculated according to the formula (percentage overlap = volume of parcel ROI within ALE sphere/total volume of parcel ROI). Any parcellation that had more than 10% of its volume within the ALE cluster was included in further analyses of our SN model.

### 2.4 Network tractography

Publicly available imaging data from the HCP were obtained for this study from the HCP database (http://humanconnectome.org, release Q3) to be utilized for structural analyses as elucidated elsewhere (Briggs, Lin, et al., 2021; Briggs, Tanglay, et al., 2021; Palejwala et al., 2021; Sandhu et al., 2021; Sheets, Briggs, Dadario, et al., 2021; Tanglay et al., 2021). Diffusion imaging with corresponding T1‐weighted images from 25 healthy, unrelated subjects was analyzed during fiber tracking analysis (subject IDs: 100307, 103414, 105115, 110411, 111312, 113619, 115320, 117112, 118730, 118932, 100408, 115320, 116524, 118730, 123925, 148335, 148840, 151526, 160123, 178950, 188347, 192540, 212318, 366446, 756055). Subjects were on average 29.5 years of age (SD = 3.8) and consisted of 13 females (52%) and 12 males (48%).

All brains were registered to the MNI coordinate space (Evans et al., 1992), wherein imaging was warped to fit a standardized brain model comparison between subjects. Tractography was performed in DSI Studio (Carnegie Mellon, http://dsi‐studio.labsolver.org) using a ROI approach to initiate fiber tracking from a user‐defined seed region (Martino et al., 2013). Specifically, a two‐ROI approach was used to isolate tracts, which included between any two cortical regions included in our model (Kamali et al., 2014). We expand on the specific parameters of our tractographic analyses below.

A multishell diffusion scheme was utilized on the diffusion scans consisting of three shells of *b*‐values equal to 1000, 2000, and 3000 s/mm^2^. *b*‐values sampled in approximately 90 directions (Sotiropoulos et al., 2013). According to DSI studio software, an automatic quality control routine assessed the *b*‐table for accuracy (Schilling et al., 2019). The in‐plane resolution and slice thickness were both 1.25 mm. Diffusion data were reconstructed at high angular resolution by utilizing the *q*‐space diffeomorphic reconstruction methodology, which in turn allows the reconstruction of the spin distribution function (Yeh & Tseng, 2011). The diffusion data were reconstructed using generalized q‐sampling imaging (GQI) with a diffusion sampling length ratio of 1.25 and an isotropic output resolution of 1 mm (Yeh et al., 2010). Restricted diffusion was quantified using restricted diffusion imaging (Yeh et al., 2017), and our tractography was completed utilizing a deterministic fiber tracking algorithm (Yeh et al., 2013). A seeding region was placed in the whole brain, and the ending regions included our individual ROIs (see Supplementary Material for individual measurements per ROI).

Voxels within each ROI were automatically traced with a maximum angular threshold of 45 degrees, and a step size of 1.5 mm was utilized. When a voxel was approached with no tract direction or a direction change of greater than 45 degrees, the tract was halted. The anisotropy threshold was selected at random according to the DSI studio software. According to recommended default settings for DSI Studio, tractography was terminated after reaching a maximum length of 800 mm, and any tracts with a tract length shorter than 1 mm were excluded. In some instances, exclusion ROIs were placed to exclude obvious spurious tracts that were not involved in the white matter pathway of interest.

### 2.5 Measuring connection strength

To quantify the strength of the connections identified within the SN across all subjects, the tracking parameters used within DSI Studio were modified such that the program would count the total number of tracts between any two ROIs based on a random seed count of 1 million. Working sequentially through ROI pairs in the network, the number of tracts between regions was recorded for each subject after fiber tractography was terminated under these new conditions.

Two different values for the strengths of the connections within the SN were calculated to identify any interindividual variability: (1) the average number of tracts across all subjects and (2) the average number of tracts across only the subjects in which the connection was identified, which ultimately excluded subjects who did not demonstrate the specified connections. Based on the average amount of tracts across *all* subjects, a laterality index (LI) was calculated according to the formula (right tract averages – left tract averages)/(right tract averages + left tract averages). Furthermore, average left and right hemisphere tract volumes were compared with the nonparametric Wilcoxon rank‐sum test (uncorrected).

The purpose of our tractographic analyses was to demonstrate the possible connections between individual parcellations in anatomically fine, precise detail that can be readily applied for further study on specific connected neural substrates as well as for clinical translation. Therefore, while differences in connection strength likely occur based on interindividual differences, tractographic analyses were able to visualize the *major* white matter connections common to most individual SN networks.

## 3 RESULTS

A total of 2220 studies were screened, and 182 full texts were assessed according to our search criteria. Ultimately, 35 studies were included in our meta‐analysis.

### 3.1 ALE regions and their corresponding parcellations

**FIGURE 1:**
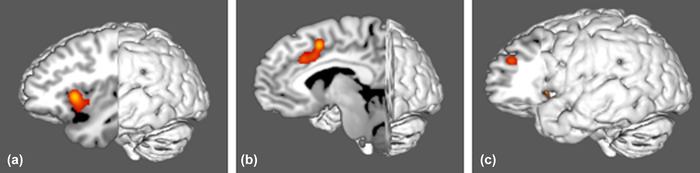
Activation likelihood estimation (ALE) of 12 task‐based fMRI experiments related to goal‐oriented attentional processing. The three‐dimensional ALE data are displayed in Mango on a brain normalized to the MNI coordinate space. (a) ALE data highlighting the insula. (b) ALE data highlighting the middle cingulate gyrus. (c) ALE data highlighting the cingulate gyrus

**FIGURE 2:**
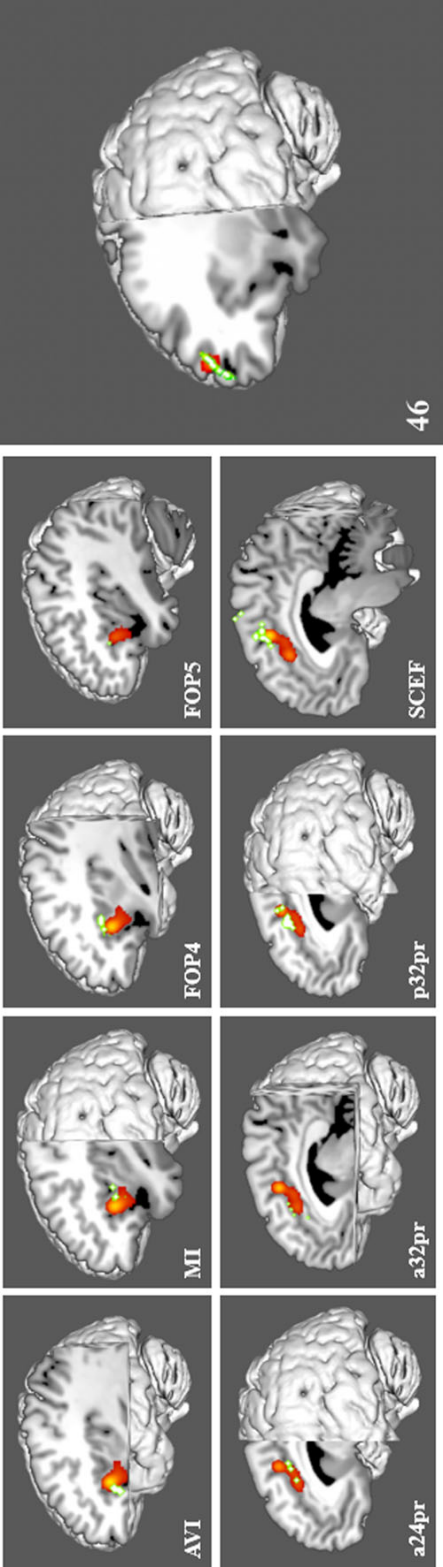
Comparison overlays between the cortical parcellation data (green) and ALE data (red) from Figure 1 in the left cerebral hemisphere. Regions were visually assessed for inclusion in the network if they overlapped with the ALE data. Parcellations included in the model of salience were identified in the insula, including AVI, FOP4, FOP5, and MI (top row); the middle cingulate gyrus, including a24pr, a32pr, p32pr, and SCEF (bottom row); and the dorsolateral prefrontal cortex, including 46 (middle). The labels indicate the parcellation shown in each panel. Abbreviations: a24pr, anterior 24 prime; a32pr, anterior 32 prime; AVI, areas anterior ventral insula; FOP4, frontal operculum 4; FOP5, frontal operculum 5; MI, middle insula; p32pr, posterior 32 prime; SCEF, supplementary and cingulate eye field

Figure 1 demonstrates the ALE of the 35 fMRI experiments included in our meta‐analysis. Highlighted areas include the bilateral frontal opercula and insulae, bilateral segments of the middle portion of the cingulate gyrus, and bilateral segments of the central portion of the dorsolateral prefrontal cortex (DLPFC). Nine ROIs were found to overlap in the fMRI data, including the anterior insula and frontal operculum areas anterior ventral insula (AVI), middle insula (MI), frontal operculum 4 (FOP4) and 5 (FOP5); middle cingulate areas anterior 24 prime (a24pr), anterior 32 prime (a32pr), posterior 32 prime (p32pr), and supplementary and cingulate eye field (SCEF); and dorsolateral prefrontal area 46. Comparison overlays between the cortical parcellations and the ALE data are shown in Figure 2.

### 3.2 Structural connections of the SN

**FIGURE 3:**
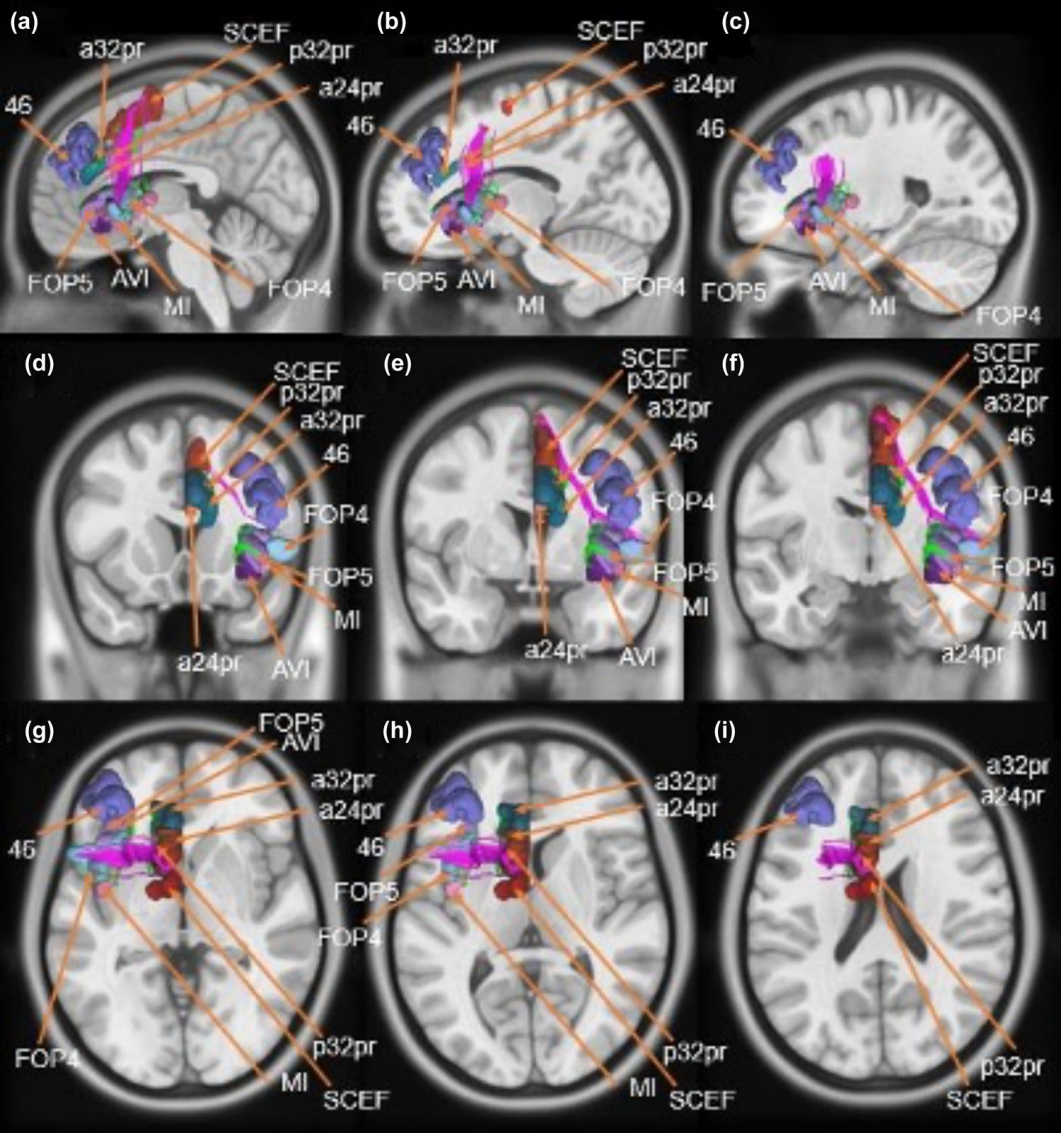
Fiber tracking analysis for the salience network (SN). T1‐weighted MR images in the left cerebral hemisphere are shown. Top row: sagittal sections from most posterior to most anterior demonstrating the frontal aslant tract (FAT) and its projections between the opercular, insular, and middle cingulate clusters of the SN. Middle row: coronal sections from medial to lateral through the parietal and occipital clusters demonstrate the FAT and the short fiber connections within the network. Bottom row: axial sections from inferior to superior provide another view of the FAT and short fiber connections within the network. Abbreviations: a24pr, anterior 24 prime; a32pr, anterior 32 prime; AVI, areas anterior ventral insula; FOP4, frontal operculum 4; FOP5, frontal operculum 5; MI, middle insula; p32pr, posterior 32 prime; SCEF, supplementary and cingulate eye field

**FIGURE 4:**
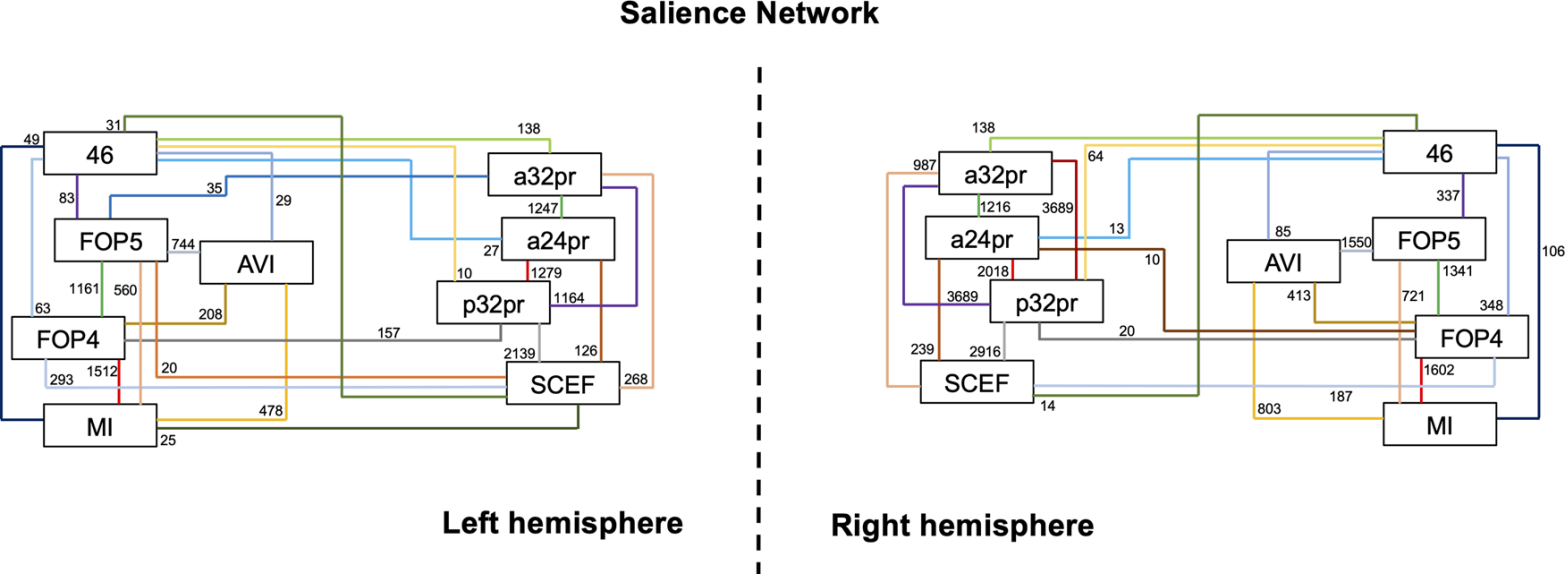
Simplified schematic of the white matter connections identified between individual parcellations of the SN during the fiber tracking analysis. Connections are labeled with the average strength measured across all 25 subjects. Abbreviations: a24pr, anterior 24 prime; a32pr, anterior 32 prime; AVI, areas anterior ventral insula; FOP4, frontal operculum 4; FOP5, frontal operculum 5; MI, middle insula; p32pr, posterior 32 prime; SCEF, supplementary and cingulate eye field

The cortical areas included in the SN can be grouped into three general clusters: an anterior insula and frontal operculum cluster (AVI, MI, FOP4, FOP5), a middle cingulate cluster (a24pr, a32pr, p32pr, SCEF), and a DLPFC cluster including a single region (46). These clusters demonstrated two main types of structural connections via the frontal aslant tract (FAT) and local association fibers, which are discussed below. The complete network model is shown in Figure 3. Furthermore, Figure 4 presents a simplified schematic of the ROIs and main structural connections included in the cortical model. Lines in this schematic represent individual connections of the SN and are labeled with their corresponding strength measured by averaging the number of tracts between ROI pairs across all subjects. Individual pairs with less than 10 connections were not included in our network model to only highlight the frequent connections that are clinically relevant.

The FAT formed nearly half of the connections (16/36, 44%) found between cortical areas within the SN. The FAT projects between the insular‐opercular cluster to the middle cingulate cluster as it courses within the white matter of the posterior frontal lobe (Figure 3). Two parcellations within the insular‐opercular cluster contribute to the FAT, areas FOP4 and MI. The fibers arise from the insula and operculum and curve gradually in the cranio‐caudal plane before reaching the middle cingulate cortex. The fibers pass close to the wall of the lateral ventricle before terminating in regions p32pr and SCEF.

Short local association fibers demonstrating a unique U‐shaped morphology (“U‐fibers”) were also identified and formed most of the connections between ROI pairs in our SN model. These U‐fibers generally have the same morphology, arising within one part of the cortex before curving 180 degrees to terminate in a part of the brain immediately adjacent to its origin. As such, these fibers represent the local connections between insular‐opercular and cingulate cortical areas nearby (discussed further in Section 4.2.2).

## 4 DISCUSSION

In this study, we constructed a detailed, cortical model of the neuroanatomic substrates involved in the SN. We identified three clusters of interconnected cortical regions that were extensively connected with both FAT fibers and short local association fibers, generally forming a large cingulate and insular‐opercular system. Specifically, the SN was found to have clusters of ROIs involved in anterior insular‐opercular, middle cingulate, and dorsolateral prefrontal cortices, which agrees well with previous work by others (Ghahremani et al., 2015; Menon & Uddin, 2010; Seeley, 2019) and that of our own team (Baker, Burks, Briggs, Conner, Glenn, Morgan, et al., 2018; Baker, Burks, Briggs, Conner, Glenn, Robbins, et al., 2018; Baker, Burks, Briggs, Stafford, et al., 2018). The white matter bundles found connecting the anterior insula and frontal operculum ROIs with the middle cingulate cortex were exclusively FAT fibers. Furthermore, our network model demonstrated strong interconnectedness within each individual cluster of ROIs via short‐local association fibers, which demonstrated a unique “U‐shaped” morphology. These U‐fibers were seen connecting possible SN nodes in the anterior insula and frontal operculum with each other as well as within nodes of the middle cingulate cortex. Together, this extensive connectivity likely supports the functional role of the SN in detecting and processing salient stimuli to guide biologically and cognitively relevant behavior as described in previous work (Menon & Uddin, 2010; Seeley et al., 2007).

Importantly, our results generally corroborate well with the findings of previous work despite utilizing grossly different methodology. However, the current work provides a more detailed cortical model of the ROIs and connections involved in the SN by utilizing combined structural and functional neuroimaging studies in the literature and by describing our results according to the detailed HCP parcellation scheme (Glasser et al., 2016). Unfortunately, the structural interconnectedness of the SN has previously remained underspecified despite both its increasing body of research over previous years and the large advancements in neuroimaging technologies made in the neuroscience community (Doyen & Dadario, 2022; Menon, 2015). Such precision and clarity of the structural white matter connectivity of the SN are necessary to better understand the essential functions of the SN according to individual neural substrates and how to navigate this region with clinical applications (Menon, 2011; Rosen et al., 2021). To understand how each of these structural substrates of the SN may support its functional relevance, it is important to first understand the speculative functions of each cortical ROI included in our model and their relative locations according to the HCP parcellation scheme.

### 4.1 Cortical regions in the SN

#### 4.1.1 T**he insular‐opercular cluster**

The insula has been described consistently within the literature as a functionally heterogeneous region, with its most anterior aspect likely forming a key hub of the SN (Menon & Uddin, 2010; Seeley et al., 2007; Uddin, 2016; White et al., 2010). Relatedly, we identified cortical areas AVI and MI that overlapped with the ALE in the anterior insula. Several functions have been ascribed to the insula, including roles in sensation and control of autonomic nervous system processes as well as human awareness, self‐recognition, time perception, and perceptual decision making (Craig, 2009; Nelson et al., 2010). The anterior insula in particular receives a variety of multimodal inputs, such as both auditory and visual information, as well as from autonomic processes, positioning it in a key position to detect and integrate both biologically and behaviorally relevant stimuli (Menon, 2015). Both areas AVI and MI in particular were only recently delineated as distinct parts of the cortex in 2016, and therefore, further information on their specific functional relevance remains scarce (Glasser et al., 2016). Anatomically, area AVI is located at the anterosuperior apex of the insula, while MI is located within the posterosuperior aspect of the short insular gyri.

Other cortical areas identified resided in the frontal opercular region according to our ALE, including areas FOP4 and FOP5. Both of these regions are located in the inferior frontal gyrus, where FOP4 is located on the inner surface of the pars opercularis, and FOP5 is located on the undersurface of the opercular part of the pars triangularis. Furthermore, they have both been described consistently within the literature as part of the SN (Elton & Gao, 2014; Menon & Uddin, 2010; Sadaghiani & D'Esposito, 2014; Seeley et al., 2007). Unfortunately, similar to AVI and MI, there is little known about areas FOP4 and FOP5, as they were described as distinct cortical areas in 2016 (Glasser et al., 2016). However, the frontal operculum is known to play a role in the initiation of language and lexical retrieval required for language learning (Li et al., 2017; Steinmetz & Seitz, 1991).

#### 4.1.2 The middle cingulate cluster

The cingulate gyrus has been divided by Brodmann into 6 regions, while the HCP has further divided it into 21 distinct regions between the anterior and posterior cingulate cortex (Baker, Burks, Briggs, Stafford, et al., 2018; Glasser et al., 2016). The anterior portion, compared to the posterior cingulate cortex, is better understood to anchor the SN (Menon, 2011; Menon & Uddin, 2010). Given that the anterior cingulate cortex contains 13 distinct regions characterized by the HCP authors, the current study highlights the imperative need for more precise anatomic network models (Baker, Burks, Briggs, Stafford, et al., 2018; Glasser et al., 2016). We specifically identified the cortical areas a24pr, a32pr, p32pr, and SCEF, which mostly overlapped with the ALE in the middle cingulate cortex and have been consistently described within the literature as forming part of the SN (Ham et al., 2013; Menon & Uddin, 2010; Seeley et al., 2007; White et al., 2010). Distinct functions have been attributed to each of these individual areas, which we describe below along with their topographical location.

Area a24pr is implicated in cognitive response selection and in word and sentence selection during language‐based tasks (Devinsky et al., 1995). Specifically, area a24pr is located in the middle cingulate gyrus, occupying the superior half of the gyrus as it extends into the inferior bank of the cingulate sulcus. In contrast, area a32pr is known to help guide behavior by evaluating motivation, anticipating outcomes, recognizing reward values, and encoding errors to influence attention allocation and motor preparation (Bush et al., 2000, 2002). Area p32pr has been implicated as part of the “cognitive division” of the anterior cingulate cortex and is involved in stimulus and response selection in tasks that require attention for linguistic and sensory information (Devinsky et al., 1995; Gasquoine, 2013). Together, compared to area a24pr, areas a32pr and p32pr are located more superiorly in the posteroinferior portion of the superior frontal gyrus. Last, SCEF is a higher‐order oculomotor center implicated in appraising all possible oculomotor behaviors for goal‐directed behavior (Stuphorn, 2015). SCEF can be seen in the posterior medial superior frontal gyrus.

Common functional themes related to the areas of the cingulate cluster include selection and appraisal of tasks (areas a24pr, a32pr, and p32pr), attention allocation (a32pr), and oculomotor control for goal‐directed behavior (SCEF; Baker, Burks, Briggs, Conner, Glenn, Robbins, et al., 2018; Baker, Burks, Briggs, Stafford, et al., 2018). These functional attributes support previous hypotheses of the SN's role in task selection as the brain switches between resting and active states of mind based on individual goals and the stimuli presented.

#### 4.1.3 The DLPFC cluster

The DLPFC was segregated into 13 distinct ROIs. While many classically relate the DLPFC to the central executive network, it is a functionally heterogeneous region and an increasingly evolved cortical area. Nonetheless, it has been previously poorly understood in terms of connectivity. In this context, we identified one parcellation within the central portion of the DLPFC, area 46, to be included in our cortical model of the SN, which also supports previous work (Schaefer et al., 2018). Unsurprisingly, area 46 has been demonstrated elsewhere with similar ALE analyses to participate in goal‐directed processes related to higher‐order cognition, such as to orient attention to and facilitating executive functions related to planning tasks (Nitschke et al., 2017; Petrides, 2005). In regard to its exact location, area 46 can be seen located along the superior frontal sulcus posteriorly, with its anterior portion located on parts of the middle frontal gyrus (Briggs, Lin, et al., 2021). It is structurally connected to several other cortical parcellations outside of the SN network as well, such as to adjacent regions of the DLPFC participating in the central executive network (Baker, Burks, Briggs, Conner, Glenn, Morgan, et al., 2018).

Importantly, this parcellation has been previously characterized by our team and many others to be likely the most successful cortical target for modulatory treatments to alleviate depressive symptoms. A recent study on veterans with treatment‐resistant major depression demonstrated that area 46 was the common target in successful treatment responders to repetitive transcranial magnetic stimulation, compared to other slightly adjacent DLPFC targets that were significantly more common in nonresponders, such as area 8AV (Rosen et al., 2021). These differences reflect that despite the close dspatial proximity of any two targets, they could modulate different networks, specifically area 46 with the SN and area 8AV with the DMN. Such clarification could improve the efficacy of large randomized controlled trials (RCT) fm that have failed to detect meaningful treatment improvements due to imprecise cortical targeting and subsequent network modulations, suggesting the need for more precise parcellation‐based network models as in this study (Moreno‐Ortega et al., 2020; Yesavage et al., 2018).

### 4.2 Structural connectivity of the SN

Improving our understanding of the precise connectivity of the SN can shed important insight into how specific neuroanatomic substrates in the SN facilitate the human ability to identify and process biologically and behaviorally relevant stimuli and how this information may differ between specific individuals. The strength and fiber type of individual connections identified between parcellations of the SN are reported in Table 2 according to deterministic tractographic analyses and are discussed in brief below.

**TABLE 2:** The strength of the connections identified between parcellations of the salience network

		**Left hemisphere**			**Right hemisphere**			Laterality index (P‐value)
Connection	Fiber Type	Number of brains	Average tracts across brains when present	Average tracts across all brains	Number of brains	Average tracts across brains when present	Average tracts across all brains	
a24pr – a32pr	U‐fiber	25	1246.72	1246.72	25	1216	1216	‐0.01 (0.97)
a24pr – AVI	FAT	3	16.67	2	0	0	0	‐1.00 (‐)
a24pr – FOP4	FAT	3	56.67	6.8	2	125	10	0.19 (0.72)
a24pr – FOP5	FAT	3	57.33	6.88	2	22	1.76	‐0.59 (0.39)
a24pr – MI	FAT	1	4	0.16	1	222	8.88	0.96 (0.33)
a24pr – p32pr	U‐fiber	25	1278.96	1278.96	25	2017.68	2017.68	0.22 (0.29)
a24pr – SCEF	U‐fiber	12	262.33	125.92	10	597.4	238.96	0.31 (0.54)
a24pr – 46	Other	3	224.67	26.96	3	111.33	13.36	‐0.34 (0.29)
a32pr – AVI	FAT	4	55	8.8	1	226	9.04	0.01 (0.98)
a32pr – FOP4	FAT	6	13.67	3.28	5	34.8	6.96	0.36 (0.42)
a32pr – FOP5	FAT	6	144.67	34.72	3	4	0.48	‐0.97 (0.09)
a32pr – MI	FAT	1	20	0.8	1	14	0.56	‐0.18 (0.81)
a32pr – p32pr	U‐fiber	2	1164.4	1164.4	25	3688.8	3688.8	0.52 (0.33)
a32pr – SCEF	U‐fiber	23	290.96	267.68	13	1898.62	987.28	0.57 (0.22)
a32pr – 46	Other	8	431.25	138	7	189.43	53.04	‐0.44 (0.52)
AVI – FOP4	U‐fiber	22	235.82	207.52	22	469.55	413.2	0.33 (0.23)
AVI – FOP5	U‐fiber	25	744.08	744.08	24	1614.92	1550.32	0.35 (0.34)
AVI – MI	U‐fiber	24	497.67	477.76	24	837.33	803.84	0.25 (0.45)
AVI – p32pr	FAT	5	16.4	3.28	2	117	9.36	0.48 (0.50)
AVI – SCEF	FAT	2	86	6.88	0	0	0	‐1.00 (‐)
AVI – 46	Other	5	146	29.2	3	706	84.72	0.49 (0.51)
FOP4 – FOP5	U‐fiber	25	1160.8	1160.8	25	1341.28	1341.28	0.07 (0.80)
FOP4 – MI	U‐fiber	25	1511.92	1511.92	25	1601.92	1601.92	0.03 (0.95)
FOP4 – p32pr	FAT	15	262	157.2	5	102	20.4	‐0.77 (0.17)
FOP4 – SCEF	FAT	16	457.63	292.88	7	668.29	187.12	‐0.22 (0.52)
FOP4 – 46	Other	6	262	62.88	9	965.33	347.52	0.69 (0.31)
FOP5 – MI	U‐fiber	22	636.36	560	20	901.4	721.12	0.13 (0.78)
FOP5 – p32pr	FAT	5	22.8	4.56	3	34.67	4.16	‐0.05 (0.93)
FOP5 – SCEF	FAT	6	82	19.68	1	6	0.24	‐0.98 (0.08)
FOP5 – 46	Other	5	414.4	82.88	9	935.56	336.8	0.61 (0.34)
MI – p32pr	FAT	4	23.5	3.76	1	4	0.16	‐0.92 (0.17)
MI – SCEF	FAT	9	68.44	24.64	4	21	3.36	‐0.76 (0.13)
MI – 46	Other	3	406	48.72	3	880	105.6	0.37 (0.62)
p32pr – SCEF	U‐fiber	25	2138.72	2138.72	25	2915.84	2915.84	0.15 (0.43)
p32pr – 46	Other	6	43.67	10.48	3	532.67	63.92	0.72 (0.24)
SCEF – 46	Other	11	70.55	31.04	3	114.67	13.76	‐0.39 (0.39)

*Note*: Two different values for strength are recorded based on the average number of tracts across all subjects versus the average number of tracts across subjects in which the connection was identified. The LI is calculated using the formula (right average – left average)/(right average + left average). Left and right average tract volumes were compared with the unpaired Wilcoxon rank‐sum test (uncorrected).

Abbreviations: a24pr, anterior 24 prime; a32pr, anterior 32 prime; AVI, areas anterior ventral insula; FAT, frontal anterior tract; FOP4, frontal operculum 4; FOP5, frontal operculum 5; MI, middle insula; SCEF, supplementary and cingulate eye field.

**TABLE 3:** Function of each cortical region of interest included in our model and their relative locations according to the Human Connectome Project (HCP) parcellation scheme

	Coordinates inMNI space				
HCP brain parcellation	*x*	*y*	*z*	Clustersize	Speculative function
AVI	−33	22	−1	2	Salience node; responsible for human awareness, self‐recognition, perceptual decision making; autonomic processes
MI	−39	7	−3	4	Salience node; responsible for human awareness, self‐recognition, perceptual decision making; autonomic processes
FOP4	−40	13	5	7	Initiation of language and lexical retrieval
FOP5	−39	22	6	3	Initiation of language and lexical retrieval; motor activity (likely more than FOP4)
a24pr	−6	10	36	4	Cognitive response selection (especially for language‐based tasks)
a32pr	−6	27	34	2	Influences attention allocation and moto preparation by evaluating stimuli according to motivation and reward
p32pr	−7	16	39	4	Stimulus and response selection (especially related to linguistic and sensory information)
SCEF	−6	9	51	6	Guides oculomotor behavior according to goals
46	−33	34	34	3	Goal‐directed behavior; key target for depression

Abbreviations: a24pr, anterior 24 prime; a32pr, anterior 32 prime; AVI, areas anterior ventral insula; FAT, frontal anterior tract; FOP4, frontal operculum 4; FOP5, frontal operculum 5; MI, middle insula; MNI, Montreal Neuroimaging Institute; SCEF, supplementary and cingulate eye field.

#### 4.2.1 The SN consists of FAT and U‐shaped association fibers

Many of the fiber bundles demonstrated in our network model were in the form of FAT fibers. Namely, ROIs in the fronto‐opercular cluster demonstrated connectivity via FAT fibers to ROIs in the middle cingulate cluster. Similar FAT connections have not been described in other cortical models of the SN, which have mostly implicated the uncinate fasciculus (Menon, 2015). This absence likely reflects the relatively nascent identification of the FAT in 2008, only 1 year after the general characterization of the SN in humans (Catani et al., 2012; Seeley et al., 2007).

While our team has been consistently incorporating preoperative tractography of the FAT for many years to attempt to reduce cognitive morbidity (Briggs, Allan, et al., 2021), such connections have been recently implicated in a variety of neurologic disorders that necessitates further study on these connections (Menon, 2011; Uddin et al., 2013). For instance, disconnecting the FAT is strongly associated with the classically described “SMA syndrome,” characterized by hemiparesis and mutism, and preservation of the FAT mostly prevents these deficits (Briggs, Allan, et al., 2021). Through a number of multinetwork interactions with higher‐order networks and the motor system along the medial frontal lobe, it is possible that the FAT is the primary fiber that facilitates the role of the SN in appropriately transitioning internal goals and thoughts into executable actions, such as with speech or motor planning (Dadario et al., 2021; Menon, 2011; Poologaindran et al., 2022). However, the current work cannot confirm this functional relevance, and our data support that possible connections exist that may facilitate these functions and require further clinical study.

The other fibers identified in the current study were short, local association fibers that took on a distinct “U‐shaped” morphology. U‐shaped segments were seen connecting each ROI within their respective clusters. U‐fibers are commonly identified across the human cerebrum, linking adjacent gyri. Compared to common diffusion tensor imaging‐based tractographic analyses used in previous studies, DSI‐based analyses with GQI‐based methodology, as employed in the current study, are better at resolving U‐shaped crossing fibers due to methodological differences in techniques (Qi et al., 2012). As such, their speculative functions may be more easily identifiable compared to the FAT. Given the short nature of these U‐fibers with closely located cortical regions, they are generally described to facilitate quick information transfer between different regions within a network, usually in a single brain lobe (Briggs, Tanglay, et al., 2021).

#### 4.2.2 Strength of connections within the SN

It is certainly the case that the structural connectivity of the SN varies to some degree between individuals, and by presenting both sets of average connection strengths, one can see how these connections can vary in the network (Table 1). For example, the connection from area a32pr to FOP5 has an average strength of 34.72 across all 25 subjects (meaning one would expect to find 34.72 streamlines using the fiber tracking algorithm discussed in the methods) versus an average strength of 144.67 in the six individuals in which the connection was identified. By reporting both numbers, we can see that while the connection between a32pr and FOP5 is relatively infrequent in the network (6/25, 24%), in the specific individuals who have such a connection, it is relatively strong. Comparatively, connections between a32pr‐SCEF were present in 23/25 subjects (92%) and therefore likely include a more common structural connection that can be identified in most SNs analyzed.

It should also be noted that we did not set a threshold for the strength that might limit the connections shown for the SN. For example, assessing the connection between MI and p32pr via the FAT, one sees that the average strength across all 25 subjects used in this study was 3.76 versus 23.5 in the four subjects for whom such a connection was identified. If we had set a threshold of an average strength of 10.0 or set a threshold related to the frequency by which we saw the connection, that is, in at least 20/25 subjects, then we would not report this connection at all. However, such a strict definition may incorrectly overlook important interindividual differences, which could provide insight into unique pathophysiological states or for clinical translation. Instead, it is more appropriate to say that the connection between MI and p32pr, while relatively weak compared to other connections in the network, still occurs infrequently in the SN, as opposed to reporting that no such connection exists between these two areas. Despite not setting a threshold, the frequency and strength associated with similar weak connections compared to what is seen between MI and SCEF raise the serious question of whether this connection is critical for the functionality of the network. Nonetheless, answering questions about what connections are more functionally “eloquent” is beyond the scope of this study and requires further information in a clinical setting.

### 4.3 Limitations and future directions

While we attempted to construct a cortical model of the neuroanatomic substrates involved in the SN with high precision by utilizing novel meta‐analytic and precise connectivity‐based analyses, the current study is not without its limitations. Similar to all meta‐analyses, findings from the current study are limited by the quality of the reported literature and may be influenced by possible publication bias. Fortunately, coordinate‐based ALE analyses employ strict cluster‐level interference algorithms that attempt to only identify statistically likely ROIs that coactivate together based on certain search parameters incorporated (Eickhoff et al., 2012, 2016; Turkeltaub et al., 2012); however, the absence of studies in the literature that report negative results precludes the inclusion and effective investigation of more regions in our analyses. A recently suggested method that may improve this limitation in the future is to include additional code that creates fictious “noise studies” to control for possible unreported results (Acar et al., 2018).

An additional benefit of utilizing coordinate‐based, meta‐analytic methodology is the ability to overcome the limitations of heterogenous topographical nomenclature often utilized in the literature for homologous brain structures. By including reported stereotactic coordinates from previous studies, more anatomically precise results can be obtained. In this context, we have been attempting to characterize all of our current findings within the HCP nomenclature given its more precise parcellation scheme (Kuiper et al., 2020; Milton et al., 2021; Sandhu et al., 2021; Sheets, Briggs, Dadario, et al., 2021). For instance, the anterior cingulate cortex and insula are known to be functionally divided within numerous subdivisions (Glasser et al., 2016), yet previous studies on the SN network continue to generally reference these regions as just general nodes in the SN. Such vague characterizations can disbar adequate hypothesis comparison between studies, limit reproducibility, and fail to provide anatomically precise information at a level of granularity that can now be utilized for clinical treatments (McCoy et al., 2021; Poologaindran et al., 2021 ). By acquiring and analyzing coordinates and then applying the subsequent results in the precise HCP parcellation scheme, our results of specific cortical regions and their unique connectivity in the SN can provide a preliminary model for further study on how this brain organization supports human functioning and to guide clinical decision making in associated cortices.

With improved information on the human connectome, the neuroscientific community has acquired improved opportunities to individualize treatment in the context of a number of pathophysiologic states. However, combined structural‐functional studies inherently face certain limitations due to commonly described concerns of tractography analyses. Surely, it is understood that ALE results provide more rigorous quantitative results than what current diffusion studies are capable of doing (Eickhoff et al. 2012, 2016; Schilling et al., 2019). For instance, as mentioned in Table 2, one could see a certain degree of individual variability within specific white matter connections by comparing the average amount of tracts in all subjects versus only subjects in which the tracts were identified. Furthermore, while this methodology has been applied by our team over numerous other cortical regions, we have found that our results are relatively robust to the number of participants usually included (i.e., averages of 80 subjects are likely similar to those of 10 subjects), and there is always some degree of individual variability with tractographic analyses (Briggs, Tanglay, et al., 2021; Palejwala et al., 2021; Sheets, Briggs, Dadario, et al., 2021). These differences are largely due to the inherent uniqueness of neural structures between individuals. Such observations should not influence one to disregard tractographic analyses but rather consider their primary importance in *qualitatively* demonstrating the nature of the more common, *major* white matter connectivity between ROIs. In particular, gathering a more complete understanding of the major structural connectivity of a region holds unique applications within the neurosurgical community when operating in this region (Briggs, Allan, et al., 2021; Burks et al., 2017; Dadario et al., 2022; Zhiqiang et al., 2022). Furthermore, variability in these structural results provides a basis for future studies to more rigorously investigate how interindividual differences in brain structural‐functional relationships related to the SN may underlie unique physiological and pathophysiological states (Barron et al., 2021). While future improvements in these analyses are surely exhilarating, it is important to interpret the results of the current study within the context of its limitations.

## 5 CONCLUSION

We present a preliminary cortical model of the neuroanatomic substrates involved in the SN. The SN comprises parcellations within the anterior insula and frontal operculum cortices that are connected with the middle cingulate cortex via the FAT. Numerous short, U‐shaped fibers were also found linking adjacent clusters of SN nodes located in the middle cingulate cortex and anterior insula. Our parcellation‐based connectivity model of the SN provides anatomically precise data that serve as an empirical foundation to be refined in future studies.

## CONFLICT OF INTEREST

Michael Sughrue is the chief medical officer, co‐founder, and shareholder of Omniscient Neurotechnology. Charles Teo is also a consultant for Aesculap and Omniscient Neurotechnology. Isabella Young is an employee of Omniscient Neurotechnology. No products related to this were discussed in this paper. No other authors report any conflict of interest.

## AUTHORS CONTRIBUTION

*Conceptualization, methodology, software, writing–original draft*: Robert G. Briggs. *Methodology, software, project administration*: Isabella M. Young. *Writing–original draft, data curation, formal analysis, writing–review and editing*: Nicholas B. Dadario. *Data curation, formal analysis, software, writing–review and editing*: R. Dineth Fonseka. *Formal analysis, software*: Jorge Hormovas. *Formal analysis, software*: Parker Allan. *Formal analysis, software*: Micah L. Larsen. *Formal analysis, software*: Yueh‐Hsin Lin. *Data curation, formal analysis, software*: Onur Tanglay. *Formal analysis, software*: B. David Maxwell. *Data curation, formal analysis, software*: Andrew K. Conner. *Data curation, formal analysis, software*: Jordan F. Stafford. *Data curation, formal analysis, software*: Chad A. Glenn. *Project administration*: Charles Teo. *Conceptualization, methodology, software, validation, project administration*: Michael E. Sughrue.

### PEER REVIEW

The peer review history for this article is available at https://publons.com/publon/10.1002/brb3.2646

## Supporting information

Supporting Information

## Data Availability

Data are available upon request to the corresponding author.
