# Supplementary material for: Parcellation‐based tractographic modeling of the salience network through meta‐analysis
Source: Brain Behav. 2022 Jun 22;12(7):e2646. doi: 10.1002/brb3.2646 (PMC9304834; doi:10.1002/brb3.2646)
Supplement: Supplementary file 1 — Supporting Information [file BRB3-12-e2646-s001.docx]

**Supplementary Material**

1.0 Tractography Extended:

Within the current manuscript, we have explained the parameters of our tractography analyses completed in DSI Studio (<http://dsi-studio.labsolver.org/>). Regarding the actual seeding methodology utilized, we expand on this further below according to an autogenerated output obtained directly from DSI Studio.

A deterministic fiber tracking algorithm was utilized(Yeh et al., 2013). For our tractography, a seeding region was placed at whole brain. Then, tracts were calculated to each individual ROI as described here according to the left hemisphere. An ending region was placed at L_a24pr (83,57,79) with a volume size of 8.5e+02 mm cubic. An ending region was placed at L_a32pr (86,45,80) with a volume size of 1.6e+03 mm cubic. An ending region was placed at L_AVI (1.1e+02,50,46) with a volume size of 7.5e+02 mm cubic. An ending region was placed at L_FOP4 (1.2e+02,62,55) with a volume size of 1.8e+03 mm cubic. An ending region was placed at L_FOP5 (1.1e+02,48,54) with a volume size of 8.1e+02 mm cubic. An ending region was placed at L_MI (1.2e+02,65,51) with a volume size of 1.7e+03 mm cubic. An ending region was placed at L_p32pr (87,60,88) with a volume size of 9.2e+02 mm cubic. An ending region was placed at L_SCEF (85,69,1.1e+02) with a volume size of 3e+03 mm cubic. An ending region was placed at L_46 (1.2e+02,37,83) with a volume size of 3.4e+03 mm cubic. The anisotropy threshold was randomly selected. The angular threshold was 45 degrees. The step size was 1.5 mm. Tracks with length shorter than 10 or longer than 200 mm were discarded. Ultimately, a total of 1000 tracts were calculated.
